# Usage of Direct Acting Oral Anticoagulants in Cirrhotic and Non-Cirrhotic Portal Vein Thrombosis: A Systematic Review

**DOI:** 10.7759/cureus.16922

**Published:** 2021-08-05

**Authors:** Sachin Gupta, Jessica Hidalgo, Balraj Singh, Aditya Iyer, Yang Yang, Alexandra Short, Sandeep Singh, Harshil Bhatt, Sorab Gupta

**Affiliations:** 1 Hospital Medicine, Tower Health Reading Hospital, West Reading, USA; 2 Internal Medicine, San Francisco de Quito University, Quito, ECU; 3 Hematology/Oncology, Saint Joseph's University Medical Center, Paterson, USA; 4 Internal Medicine, Washington Hospital Center, Washington DC, USA; 5 Internal Medicine, Thomas Jefferson University Hospital, Philadelphia, USA; 6 Library Services, Tower Health Reading Hospital, West Reading, USA; 7 Internal Medicine, Indiana University School of Medicine, South Bend, USA; 8 Internal Medicine, Goshen Hospital, Goshen, USA; 9 Oncology, Bronx Care Health System, New York, USA

**Keywords:** portal vein thrombosis, direct-acting oral anti-coagulants, newer oral anticoagulants, portal thrombosis, anticoagulation, cirrhosis

## Abstract

Thrombosis of the portal vein (PVT) is generally seen in the setting of liver cirrhosis and to a lesser extent in the absence of cirrhosis. There is no clear guidance in relation to approaching treatment with anticoagulation in this condition. The professional societies and guidelines recommend treatment with traditional anticoagulation like low-molecular-weight heparin and vitamin-K antagonists in patients presenting with acute portal vein thrombosis. There is no clarity in relation to treatment in the setting of chronic PVT and in patients with cirrhosis. Also, the role of direct-acting oral anticoagulants (DOACs) that are becoming a preferred choice for anticoagulation for various other indications is not clear in the case of PVT. There are a very few studies in the medical literature that have investigated the role of DOACs in patients with PVT in different settings. Thus, we performed a systematic review of the literature to study the use of DOACs in PVT in patients with and without cirrhosis. The results of the available studies show that DOACS appears to be a promising choice for the treatment of patients with PVT. The availability of more data in the future along with better availability of the approved reversal agents for various DOACs is expected to make DOACS a preferred choice for the clinicians to treat patients with PVT.

## Introduction and background

Thrombosis of the portal vein (PVT) is common in patients with cirrhosis with prevalence ranging from 5% to 24% in studies that utilized ultrasonography to 6-64% in autopsy series [1]. PVT is seen in patients without liver disease as well, but the exact prevalence in that population is not clear [2,3]. Besides inherited or acquired prothrombotic states which are the major risk factors for PVT in both patients with previously healthy liver and cirrhosis [4-7], other risk factors include primary and secondary hepatobiliary malignancies, intra-abdominal infectious or inflammatory diseases, and myeloproliferative disorders [8]. However, in a significant percentage, no predisposing factors are identified [8].

Based on the acuity of presentation, PVT is classified mainly as acute or chronic. Patients with acute onset of PVT may have sudden onset of partial or complete occlusion of the portal vein leading to presentation with a wide spectrum from being silent or mild abdominal pain to catastrophic events like variceal bleeding and intestinal ischemia and infarction [9]. Patients with acute PVT, where the clot does not resolve (with or without treatment) go on to develop chronic PVT. As patients with chronic PVT generally develop collateral blood vessels bypassing the area of obstruction thus, such patients are generally asymptomatic and discovered incidentally or may present with symptoms and sequelae related to chronic complications of chronic PVT including portal hypertension and portal cholangiopathy [10-12].

As per current guidelines, anticoagulation is generally recommended for patients with acute PVT who do not have cirrhosis [3,7]. Based on studies that have shown partial or complete recanalization of the portal vein in up to 90% of the patients and subsequently improved portal vein patency in the patients who were started on anticoagulation immediately, anticoagulation is recommended in these patients to allow recanalization and to prevent the extension of the clot and other chronic sequelae like intestinal infarction and portal hypertension [4,8].

While there is evidence of the benefit of using anticoagulation in acute PVT, its role in patients with chronic PVT is unclear. Patients with chronic PVT and cirrhosis are at higher risk of bleeding due to varices [9], thus, necessitating screening for esophageal varices before initiation of anticoagulation in such patients [3]. Also, while previous studies have shown no association between PVT and progression of hepatic decompensation in patients with cirrhosis over time [10], there are reports based on animal models and case reports that PVT re-canalization may lead to improvement in liver functions over time [11,13]. At the same time based on expert opinion, it is recommended to use anticoagulation for the treatment of PVT in patients with cirrhosis who are candidates for liver transplantation [12,14] as the presence of advanced PVT is associated with surgical complexities and poor postoperative outcomes after transplantation surgery [15-17].

Similarly, the understanding regarding the issue of choosing an appropriate anticoagulation agent for the patients in whom anticoagulation is indicated is evolving. As per the guidelines from the American College of Gastroenterology (ACG) and the American Association of Study of Liver Diseases (AASLD), initiation of treatment with unfractionated heparin or low molecular weight heparin (LMWH) should be considered when a decision is made to start anticoagulation in patients with portal vein thrombosis [3,7]. This is followed by maintenance of anticoagulation on LMWH or warfarin for the rest of the duration of planned therapeutic anticoagulation [3,7]. Over the past several years, many direct oral anticoagulant agents like anti-factor Xa inhibitors (such as apixaban, edoxaban, rivaroxaban) and factor II inhibitors such as dabigatran and others have come into prominence and have been approved and being utilized for several indications like stroke prophylaxis in atrial fibrillation, DVT prophylaxis in orthopedic surgical patients to the treatment of patients with thromboembolic conditions like deep vein thrombosis and pulmonary embolism [18]. Easy accessibility of these agents and since these do not generally need regular monitoring as in case of warfarin or daily or twice daily injections as in case of LMWH accompanied by somewhat better safety profile have made direct oral anticoagulants (DOACs) a popular choice for anti-coagulation amongst clinicians [18]. While these agents have been utilized for other indications experience with them in the setting of portal vein thrombosis in patients with and without cirrhosis is limited. Few studies have investigated the use of DOACS in patients with portal vein thrombosis but most of the evidence in literature is based on published case reports and small retrospective studies only. Moreover, guidelines from ACG suggest caution while using DOACS in this setting as their absorption might be reduced in the setting of intestinal edema, thus necessitating their monitoring, which offsets one of the major advantages over warfarin [7].

Thus, we planned to perform a systematic review of the literature to evaluate the usage and performance of DOACs in the setting of portal vein thrombosis in patients with and without cirrhosis.

## Review

Materials and methods 

Protocol

For this systematic review, we used the Preferred Reporting Items for Systematic Reviews and Meta-Analysis (PRISMA) guidelines.

Eligibility Criteria and Study Selection

We included original articles, case reports, case series, and abstracts published in the last 10 years in English literature. We excluded papers that did not fulfill the aim of our study. After screening the studies, we included papers with the following inclusion criteria: (i) studies involving human subjects >18 years old, (ii) confirmed diagnosis of PVT with/without cirrhosis, (iii) treatment with one or more DOACs agents. Studies/case reports which did not specify the outcomes of treatment were not included.

Database and Search Strategy

We performed a literature review using three electronic databases: PubMed, EMBASE, and Cochrane Central Register of Controlled Trials. The search terms used were: (direct-acting oral anticoagulants) OR (direct-acting oral anticoagulants) OR (direct oral anticoagulants) OR (DOAC) OR (non-vitamin K antagonist) OR (factor Xa Inhibitors) OR (apixaban) OR (rivaroxaban) OR (Eliquis) OR (Xarelto) OR (edoxaban) OR (factor II inhibitors) OR (direct thrombin inhibitor) OR (dabigatran) OR (Pradaxa) AND (Portal vein thrombosis) OR (PVT). The most recent search was conducted on June 17, 2021, which showed a total of 713 records: PubMed (77), EMBASE (618), and Cochrane Central (18).

Data Extraction and Analysis

Initial screening of the articles was performed by three authors (SG, JH, AI) for eligibility. Subsequent abstract and manuscript review and data extraction were performed by the other three authors (YY, BS, SS). Data extraction for case reports/case series included authors’ last name, year of publication, demographic information of the patient (age and gender), setting of PVT (acute or chronic), underlying condition (cirrhosis or no cirrhosis, other risk factors for PVT), type of DOAC used and dosage (if available), duration of anticoagulation (if available), bridging agent for anticoagulation used (if available), outcomes and adverse events. For the original studies, we extracted information like author’s last name, year of publication, type of study, number of participants in the study and comparison group (if available), type of DOAC agent used, outcomes (primary/ secondary/adverse events - if reported). Discrepancies amongst the authors were resolved by another author (SoG). Article screening and determination of eligibility were performed as per PRISMA 2009 guidelines.

Results 

**Figure 1:**
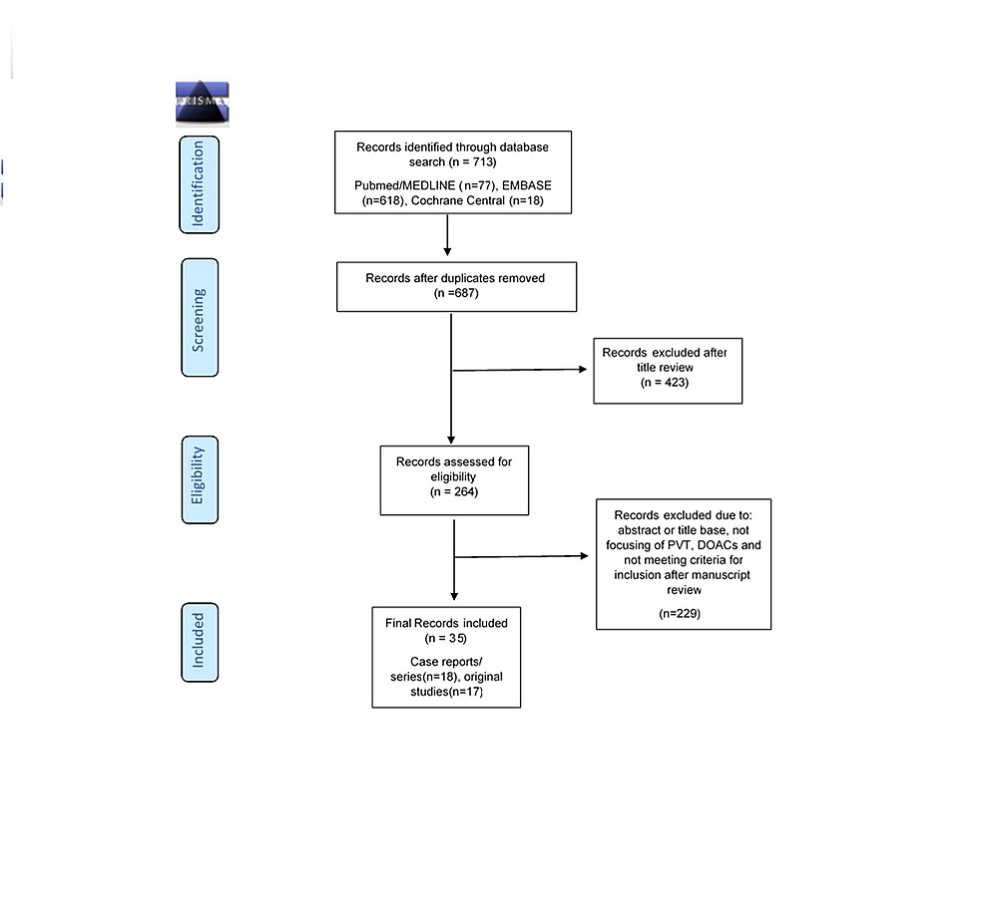
Shows the results of the study using PRISMA Flow Chart n: number of records/articles; Cochrane Central: Cochrane Central Register of Controlled Trials.

Figure 1 shows the results of the study using a PRISMA flow chart.

Based on the inclusion/exclusion criteria, a total of 713 records were identified, of which 26 studies were duplicated. After final analyses, a total of 35 records were included in this systematic review of which 18 were case reports and 17 were original studies.

Of the 17 studies, 13 studies were full manuscripts and 4 were abstracts presented at various conferences/meetings. The majority (12 out of 17) of studies were retrospective in nature. Other study designs included four prospective studies and one randomized controlled trial. A total of 18 case reports and series including 19 cases met the criteria for inclusion in this systematic review. Out of these 18 case reports 15 were published as full manuscripts in the journals and 3 were presented as case reports in the various meetings.

Table 1 shows the author, year, type of study, study group, control group, primary and secondary outcomes, and adverse outcomes of the original studies [19-36].

**Table 1:** Characteristics of studies included PVT: portal vein thrombosis; A/C: anticoagulation; TEG: thromboelastography; DOACs: direct oral anticoagulants; N/A: not applicable; HCV: hepatitis C virus; GI: gastrointestinal; VTE: venous thromboembolism.

#	Author (ref)	Year	Type of study	Study population	Study group	Control group	Primary outcomes	Secondary outcomes	Adverse outcome
1	Ai et al. [19]	2020	Prospective cohort	Chronic PVT in cirrhosis	Rivaroxaban (n=26), dabigatran (n=14)	No A/C (n=40)	28.2% complete/partial recanalization in the study arm at 6 months (P<0.05).	Improvement in total bilirubin levels, Child-Pugh scores, TEG coagulation index (p<0.05).	No statistically significant difference between the DOACs group and the control group in cases of bleeding (p>0.05).
2	Alaber et al. [20]	2019	Retrospective/meeting abstract	Adults with PVT	Warfarin, enoxaparin, apixaban, fondaparinux, rivaroxaban	Adults who had a diagnosis of PVT and did not get A/C	Cases on A/C had 1.18 times higher odds of bleeding when compared to controls who did not receive A/C.	Newer agents were less likely to increase bleeding when compared to older agents.	N/A
3	Chae [21]	2019	Prospective randomized comparative study/meeting abstract	PVT in cirrhotic patients	Dabigatran (n=3)	Warfarin (n=2)	The thrombolytic effect was not seen in any group.	N/A	N/A
4	De Gottardi et al. [22]	2017	Retrospective	Patients with splanchnic vein thrombosis (with/without cirrhosis) - included patients with PVT	Rivaroxaban, apixaban, dabigatran	N/A	DOACs are used in patients with portal vein thrombosis, Budd-Chiari syndrome, or cirrhosis.	N/A	Major bleeding (n=2; DOAC stopped), one major bleeding in the cirrhotic group, recurrent PVT (n=1).
5	Hanafy et al. [23]	2016	Randomized control trial	Acute non-neoplastic HCV-associated PVT	Rivaroxaban 10 mg BID (n=40)	Warfarin (n=40)	In the rivaroxaban group, the resolution of PVT was in 85% of the patients. In the warfarin group, the resolution of PVT was 45% of the patients.	Improved short-term survival - 20.4±2.2 months in the study arm compared to 10.6±1.8 months in the control arm.	Complications like severe upper GI bleed, hepatic decompensation, progression to mesenteric ischemia, recurrence, and death in the control group. None in the experimental group.
6	Hum et al. [24]	2016	Retrospective	Cirrhosis with all indications for anticoagulation including PVT	Subgroup with PVT - Rivaroxaban, apixaban (n=4)	Enoxaparin, warfarin (n=3)	Recurrent thrombosis occurred in one patient receiving DOAC and one patient receiving another anticoagulation.	N/A	Total bleeding events were similar in the two groups with lesser major bleeding in the DOAC group.
7	Intagliata et al. [25]	2016	Retrospective	Cirrhosis and splanchnic thrombosis	Rivaroxaban, apixaban (n=12)	Traditional A/C with warfarin and LMWH (n=6)	No statistical difference between therapeutic and prophylactic dosing between groups.	N/A	Similar rates of major and minor bleeding in the two groups.
8	Ilcewicz et al. [26]	2020	Retrospective	PVT with/without cirrhosis	DOACs (n=13)	Warfarin (n=20)	Four episodes of treatment failure in the warfarin group. None in DOAC (p<0.001).	NA	One bleeding event in the warfarin group (p<0.001).
9	Janczak et al. [27]	2017	Prospective cohort	Non-cirrhotic, atypical sites including PVT	Rivaroxaban and apixaban (N=16)	Enoxaparin for PVT(N=13) DOAC for typical location VTE (N=352)	Rivaroxaban and apixaban are effective and safe in patients with venous thrombosis of atypical locations.	N/A	No major difference in bleeding rate.
10	Mahoro et al. [28]	2019	Retrospective	Non-cirrhotic PVT	DOAC (n=94)	LMWH or vitamin K antagonist (n=67)	NA	NA	Similar rates of major bleeding in the two groups.
11	Nagaoki et al. [29]	2018	Retrospective cohort	Cirrhosis and chronic PVT with initial two-week treatment with danaparoid	Edoxaban (n=20)	Warfarin (n=30)	Reduction in volume of PVT in edoxaban arm compared to increase in volume in warfarin arm at six months.	N/A	No difference in clinically significant GI bleeding in the two groups.
12	Naymagon et al. [30]	2020	Retrospective	Non-cirrhotic PVT	Rivaroxaban (n=65), apixaban (n=20), dabigatran (n=8)	Warfarin (n=108), enoxaparin (n=70), fondaparinux (n=2)	Resolution rate - dabigatran (75%), apixaban (65%), rivaroxaban (65%), enoxaparin (57%), warfarin (31%).	Recanalization rates are higher in DOACs compared to warfarin but similar to enoxaparin.	DOACs had less major bleeding compared to warfarin and lower but not statistically significant portal hypertensive symptoms.
13	Naymagon et al. [31]	2020	Retrospective	IBD-associated PVT	DOACS (n=23)	Warfarin (n=22), Enoxaparin (n=13)	Resolution rate-DOACS (96%), warfarin (55%)	DOACs group needed a shorter course of anticoagulation (median −3.9 vs 8.5 months).	Incidence of gut ischemia, portal hypertension, major bleeding, and death rare and not reported in DOACs group.
14	Scheiner et al. [32]	2018	Retrospective	Non-malignant PVT	Total-10, edoxaban (n=4), apixaban (n=3), rivaroxaban (n=2), dabigatran (n=1)	Traditional A/C (n=12); no A/C (N=39)	Favorable outcomes with DOACs with regression/resolution of thrombus in 20% of patients and stability or non-progression in 80%.	NA	One bleeding episode in the DOAC group.
15	Walker et al. [33]	2019	Retrospective cohort	PVT with cirrhosis	Apixaban (n=82)	N/A	Complete or partial recanalization in 67.9%.	N/A	Major bleeding – 11%; minor bleeding −4%
16	Naymagon et al. [35]	2020	Retrospective	History of intra-abdominal surgery within 3 months prior to PVT diagnosis	DOACs (n=35)	No anticoagulation (n=12), Warfarin (n=31), Enoxaparin (n=29)	DOACs were associated with a complete radiographic resolution rate of (77%), enoxaparin (69%), warfarin (45%), and no anticoagulant (17%).	N/A	N/A
17	Pannach et al. [36]	2017	Prospective/meeting abstract	Acute or chronic splenic vein thrombosis with 65.2% with PVT (n=15)	Rivaroxaban	N/A	Early initiation of rivaroxaban led to fast clot resolution in most cases. Late initiation led to stable clot burden only.	N/A	Ten patients had a total of 19 bleeding complications during treatment with rivaroxaban, where 5 episodes were of major bleeding

Table 2 shows the author, year of publication, age, gender, acute/chronic, cirrhosis, predisposing factors, DOAC, dosage, bridging agent, outcome, and adverse events of the case report included [36-51].

**Table 2:** Characteristics of case report included F: female; M: male; DOAC: direct oral anticoagulants; IV, intravenous; PVT: portal vein thrombosis; BID: twice a day.

#	Author/ref	Year	Age/gender	Acute/chronic	Cirrhosis	Predisposing factors	DOAC	Dosage	Bridging agent	Duration	Outcome	Adverse events
1	Pannach et al. [36]	2020	30/F	Acute	No	Protein C, S, antithrombin deficiency	Rivaroxaban	Not specified	None	Not specified	No recurrence of acute mesenteric ischemia	None
2	Arai et al. [37]	2019	51/M	Acute	No	Intraabdominal infection and surgery	Edoxaban	60 mg/day	IV Heparin	7 months	Resolution of PVT on imaging	None
3	Eto et al. [38]	2019	70/M	Chronic	Yes	Cirrhosis	Edoxaban	Not specified	Antithrombin III	4 months	Complete resolution	None
4	Fujiwara et al. [39]	2020	70/M	Chronic	No	Rectal cancer and chemotherapy	Apixaban	10 mg BID for 7 days followed by 5 mg BID	None	8 months	Complete resolution	None
5	Zhou et al. [40]	2017	38/M	Acute	No	Protein S deficiency	Rivaroxaban	Not specified	None	Not specified	Improvement in gastroesophageal varices	None
6	Hayashi et al. [41]	2020	33/M	Acute	No	Noncirrhotic extra-hepatic porto-systematic shunt (NCPSS)	Edoxaban	Not specified	Danaparoid sodium and antithrombin III intravenous	10 months	Resolution of intrahepatic 4 part of portal vein thrombus at 3 months	None
7	Lenz et al. [42]	2014	63/F	Acute	Yes	Cirrhosis	Rivaroxaban	10 mg daily	None	Continuous	Complete recanalization	None
8	Martinez et al. [43]	2014	50/M	Acute	Yes	Cirrhosis	Rivaroxaban	20 mg daily	IV heparin	6 months	Complete resolution	None
9	Nguyen et al. [44]	2016	44/M	Chronic	No	Crohn`s disease	Rivaroxaban	Not specified	None	Not specified	Improvement in symptoms	None
10	Nery et al. [45]	2017	28/F	Acute	No	Oral contraceptive use	Rivaroxaban	15 mg BID for 3 weeks and then 20 mg daily	IV heparin followed by enoxaparin	>6 months	Partial recanalization	None
11	Obata et al. [46]	2019	38/M	Acute	No	Nephrotic syndrome	Edoxaban	30 mg PO daily	None	2 months	Complete resolution	None
12	Pannach et al. [47]	2013	56/M	Acute	No	Hemochromatosis	Rivaroxaban	20 mg daily	None	4 weeks	Complete resolution	None
13	Randhawa et al. [48]	2019	63/M	Acute	No	Positive lupus anticoagulant	Rivaroxaban	Not specified	Heparin	6 months	Resolution of presenting symptoms	None
14	Xu et al. [49]	2019	53/M	Acute	Yes	Cirrhosis	Rivaroxaban	10 mg daily	Enoxaparin	5-6 months	Complete recanalization	None
15	Yang et al. [50]	2019	52/F	Acute	No	Intraabdominal infection	Rivaroxaban	20 mg/day	None	2 months	Complete resolution of thrombus	None
16	Yang et al. [51]	2016	63/M	Chronic	Yes	Cirrhosis	Rivaroxaban	15 mg BID for 3 weeks and then 20 mg daily	None	6 months	Complete resolution	None
17	Iida et al. [52]	2020	67/M	Acute	No	Intra-abdominal surgery	Edoxaban	60 mg daily	Heparin	Not specified	Not specified	Cerebellar haemorrhage
18	Iida et al. [52]	2020	67/M	Chronic	Yes	Laparoscopic microwave coagulation for liver cancer	Edoxaban	60 mg daily	Enoxaparin	Not specified	Not specified	Cerebellar haemorrhage
19	Toyoda et al. [53]	2021	79/M	Acute	Yes	Liver cirrhosis and intra-abdominal surgery	Edoxaban	30 mg daily	None	Long term (>4 years)	PVT was markedly shrunk and patency of the portal vein trunk improved	None

Discussion

The role and the safety of DOACs in patients with cirrhosis and other liver diseases are not well established. ​The new guidelines and published expert opinions have remained conservative regarding the use of DOACs, advising continued reliance on LMWH and vitamin K antagonists (warfarin). Similarly, there are very few studies that investigated the usage of DOACs in patients with PVT in the absence of cirrhosis. A study using a large database suggests that anticoagulation increases the chances of bleeding in patients with cirrhosis and PVT but in general newer anticoagulants have a lower incidence of bleeding compared to traditional anticoagulants like warfarin and LMWH [20]. This study included fondaparinux amongst the newer anticoagulants in addition to apixaban and rivaroxaban [20]. Similarly, other studies have reported no significant difference in the incidence of bleeding or hepatotoxicity in the group of patients with cirrhosis who received DOACs for indications like PVT in comparison to the traditional anticoagulation [24,25]. Overall, DOACs were associated with fewer episodes of major bleeding [24]. A study involving both cirrhotic and non-cirrhotic patients with PVT showed better outcomes in patients treated with DOACs compared to warfarin in terms of treatment failure and adverse events [26]. Thus, the use of DOACs for the treatment of PVT is evolving but remains contentious. Hereby, we aim to summarize the findings of the various studies that investigated the usage of DOACs in patients with PVT with and without cirrhosis.

DOACS in PVT With Cirrhosis

One randomized control trial studied rivaroxaban in comparison with warfarin in acute PVT patients with non-neoplastic Hepatitis C virus (HCV) related compensated cirrhosis [23]. In this study, the experimental arm (n=40) received rivaroxaban 10 mg twice a day and the control arm (n=40) received warfarin. This study included patients with compensated cirrhosis (Child class A-B) with a mean age of 43.2±3.8 years. All the patients were bridged with enoxaparin at a dose of 1 mg/kg every 12 hours for three days prior to switching to rivaroxaban or warfarin. The experimental arm showed rapid improvement with resolution of the clot in 85% (n=32) of the subjects within 2.6±0.4 months. It also showed delayed partial resolution after 6.7± 1.2 months in the other six patients while only 45% of the subjects in the control arm showed resolution [23]. Also, the study showed improved short-term survival (20.4±2.2 months) in the experimental arm compared to (10.6±1.8 months) in the control arm [23]. The study arm did not have any adverse events reported while adverse events such as severe upper GI bleed (43.3%), hepatic decompensation (22.5%), progression to mesenteric ischemia (12.5%), recurrence of PVT (10%), and death (10%) were reported in the control group [23]. Anticoagulation was continued for a period of one to two months after the complete recanalization of the portal vein depending on the original location and extent of the thrombus. For the patients with hereditary thrombophilia, partial or delayed recanalization longer therapy of four months after partial or complete recanalization was chosen [23].

Another interventional prospective cohort study by Ai et al. studied the effects of anticoagulation with agents like rivaroxaban (n=26) and dabigatran (n=14) versus no anticoagulation (n=40) in patients with chronic PVT and in patients with cirrhosis [19]. This study included patients with chronic PVT in the age group 18-75 years and a prospective study cohort method was used to divide patients into DOACs group and control group [19]. In the experimental arm, rivaroxaban 20 mg once daily was the preferred drug and for patients with Child-Pugh grade (B) or (C) dabigatran 150 mg twice daily was used as they were considered unsuitable for treatment with rivaroxaban [19]. This study showed promising results with complete and partial recanalization rates of 12.8% in three months and 28.2% in six months in the DOAC group which were significantly higher than the control group with no treatment (*p*<0.05) [19]. The DOAC group also showed improvement in total bilirubin level, Child-Pugh scores, and TEG coagulation indices compared to the control arm [19]. Interestingly, despite anticoagulation, no significant difference was noted in the incidence of bleeding in the two arms [19]. This study did not include patients with a history of moderate to severe esophageal varices, active bleeding within three months, systemic malignancy, pregnancy, lactation, thrombocytopenia (platelet count <50 × 10^9^/L), or having abnormal coagulation profile. Also, patients on other anticoagulants, anti-platelet agents, and having both transaminases(>2 times of upper limit of normal) and Child-Pugh grade B or C were not included [19]. Another small prospective randomized comparative study to compare the thrombolytic effect of dabigatran (n=3) versus warfarin (n=2) in patients with cirrhosis and chronic PVT showed no thrombolytic effect in either group [21]. The investigators are planning a larger study involving 50 subjects and hypothesize negative effects related to the organization of the thrombi in patients with Chronic PVT [21].

Nagaoki et al. compared the effectiveness of Edoxaban (n=20) with warfarin (n=30) in patients with cirrhosis-associated chronic PVT [29]. The retrospective cohort of patients with cirrhosis-associated chronic PVT was initially treated with a course of danaparoid for a period of two weeks and then switched to either edoxaban or warfarin. The dose of Edoxaban used was 60 mg per day (30 mg per day if creatinine clearance between 30 and 50 ml/min, body weight ≤60 kg, and on concurrent P-glycoprotein inhibitor). In patients on warfarin goal of INR between 1.5 and 2.0 was maintained. The study showed a reduction in the volume of PVT in the Edoxaban arm at six months (from 1.42 cm^3^ to 0.42 cm^3^). On the contrary, there was an increase in the volume of PVT in the warfarin arm (from 1.73 cm^3^ to 2.85 cm^3^). There was a slightly higher but not statistically significant rate of GI bleeding in the Edoxaban arm (15%) compared to the warfarin arm (7%) (p=0.335) [29].

Usage of apixaban in patients with PVT and cirrhosis was evaluated in a retrospective study (n=82), which showed effectiveness in the form of complete/partial recanalization (67.9%) [33]. A lower dose of apixaban 2.5 mg twice daily was used in 87% of the patients while 5 mg twice daily was used in about 10% [33]. Apixaban had to be discontinued in 11 (13.4%) patients due to major GI bleed out of which one patient died [33]. At the same time there are some case reports that have shown usage of various DOACs in the setting of acute and chronic PVT in patients with cirrhosis [38,42,43,49,51-53] with positive outcomes in most but an incidence of intracranial hemorrhage in one case after usage of rivaroxaban [52].

DOACS in PVT Without Cirrhosis

While there are some case reports that report usage of DOACs in the setting of PVT without cirrhosis, we were able to find a few original studies and no randomized clinical trial on this subject.

A prospective study assessed the outcome of DOACS, specially Xa inhibitors like rivaroxaban and apixaban for the treatment of venous thromboembolism of atypical locations using a prospectively collected registry [27]. The study followed patients who were treated with anticoagulation (DOACs vs enoxaparin and warfarin) for acute venous thromboembolism of typical locations (VTE-TL, i.e., DVT of extremities and pulmonary embolism) versus atypical locations (VTE-AL, e.g., splanchnic veins including portal veins) [27]. Overall, the rate of recurrent VTE, minor/major bleeding, and death were not statistically different in the group with venous thromboembolism of atypical locations (including portal venous thrombosis) who received DOACs in comparison to enoxaparin [27].

Nayamagon et al. have published different studies comparing the effectiveness of various DOACS with traditional anticoagulants like warfarin, enoxaparin, and fondaparinux in non-cirrhotic PVT [30] and IBD-associated PVT [31], respectively. In a study, comparing rivaroxaban (n=65), apixaban (n=20), dabigatran (n=8) with warfarin (n=108), enoxaparin (n=70), and fondaparinux (n=2) in non-cirrhosis-associated PVT patients showed higher effectiveness of DOACs [dabigatran (75%), apixaban (65%), rivaroxaban (65%)] in terms of complete radiological resolution (CRR). The enoxaparin group also showed a comparable response of 57% (CRR) but was much lower in the warfarin group (31%) [30]. Another study by the same author, investigated DOACs(n=23) in comparison to agents like warfarin (n=22), enoxaparin (n=13) in inflammatory bowel disease-associated PVT [31]. The study showed much higher CRR in the DOAC group (96%) compared to the warfarin group (55%). Also, treatment with DOACs warranted a shorter duration of anticoagulation (median-3.9 months) compared to warfarin (median 8.5 months) [31]. Both the studies reported a much lower incidence of complications like major bleeding in the DOAC group compared to the warfarin group [30,31]. Another recent study investigated the effect of DOACs in patients having PVT within three months after an intra-abdominal surgery in comparison to no anticoagulation and traditional anticoagulants like warfarin and enoxaparin [34]. This study showed that the usage of DOACs was associated with a much higher rate of resolution of PVT. The rate of CRR was 77% in the DOACs group, while it was 69% for enoxaparin, 45% for warfarin, and 17% for no anticoagulation [34]. The study showed a significantly low hazard ratio for complete radiological resolution in the groups with enoxaparin, warfarin, and no anticoagulation with DOACs as the reference [34]. Another prospective study showed early initiation of rivaroxaban in patients with splanchnic vein thrombosis including PVT which led to early clot resolution in most cases [35]. While with a delayed initiation clot burden was stabilized but a resolution could not be achieved [35].

Another study (n=10) reported favorable outcomes of anticoagulation with DOACs in patients with non-cirrhotic, non-malignant chronic portal vein thrombosis with regression/resolution of thrombus in 20% of patients and stability/non-progression in 80% which was considered favorable as recanalization was not possible given chronic PVT with cavernous transformation [32]. One out of 10 patients treated with DOACs had a bleeding event while none was reported in the group that received traditional anticoagulation [32]. Similarly, there are some case reports which report positive outcomes with various DOACs like apixaban, rivaroxaban, and edoxaban in patients with acute and chronic PVT in patients without cirrhosis [36,37,39,41,44-48,50]. These case reports had utilized different dosages of the DOACs and for the variable duration as well. Also, there is no consistent utilization of bridging parenteral anticoagulation in the various case reports and studies. While most of the case reports have shown positive outcomes in terms of clot resolution and symptoms; a series reported cases of intracranial hemorrhage after utilization of rivaroxaban [52].

A retrospective study by De Gottardi et al. evaluated outcomes of patients with splanchnic vein thrombosis and cirrhosis who were treated with direct-acting oral anticoagulants [22]. They reported out of 58 patients with splanchnic vein thrombosis in the absence of cirrhosis who were treated with DOACs, 65% (n=38) had portal vein thrombosis and most of the patients were treated with rivaroxaban (~84%, dose range 10-20 mg), followed by apixaban (10%, dose range 5-10 mg), and dabigatran (5%, dose range 150-220 mg). Similarly, out of the 36 patients with both cirrhosis and splanchnic vein thrombosis, 61% (n=22) had portal vein thrombosis and a comparable percentage of patients received rivaroxaban (83%, dose range 5-20 mg), apixaban (11%, dose range 2.5-10 mg), and dabigatran (5%, dose range 110-220 mg) [22]. This study identified that the median daily dose of DOACs chosen for patients with cirrhosis was 25% lower than the dose chosen for patients without cirrhosis. This study did not present the outcome data exclusively for the subgroup with PVT but overall, no cases of mortality were reported. Major bleeding was reported in total 3 out of total 96 patients (3%) and DOAC had to be stopped in 7 (7%) of the patients mainly for events like minor bleeding and side effects like dizziness and disorientation, etc. In one patient with cirrhosis, DOAC was replaced with LMWH as the patient experienced recurrent portal vein thrombosis [22].

Based on the studies, it appears that DOACs appear to be a reasonable option for treating portal vein thrombosis in patients both with and without cirrhosis. There is a wide heterogeneity regarding choosing an appropriate agent for a situation, along with the dosage, duration, and parenteral bridging anticoagulation used. As most of the original trials for DOACs for situations like Atrial fibrillation and DVT/PE, etc., did not include patients with advanced cirrhosis; it makes it further difficult to find an appropriate dose of a particular DOAC agent. In the only published randomized controlled trial comparing rivaroxaban to warfarin in patients with PVT and cirrhosis [23], a lower than usual dosage of rivaroxaban (10 mg twice daily) was utilized. The dose of 10 mg twice a day for rivaroxaban is lower than the standard dose for acute DVT/PE, i.e., 15 mg twice daily for three weeks followed by 20 mg once daily after that. Other prospective studies preferred dabigatran over rivaroxaban in patients with cirrhosis with Child-Pugh grades B and C [19]. For bridging, usage of parenteral agents was infrequent and inconsistent though reported in some case reports. Despite apixaban being a common choice for anticoagulation amongst clinicians, at present studies evaluating its role in PVT with or without cirrhosis are limited. There are some studies that have been registered in the Cochrane Central Register of Controlled Trials which might add to the knowledge once completed. Some published case reports have specified usage of a DOAC despite not specifying information on outcomes and adverse events; thus, highlighting the comfort level of the clinical community to choose a DOAC as a go-to agent for anticoagulation unless obvious contraindicated.

Ease of reversibility of warfarin and limited availability of antidotes for DOACs might be a factor at present to discourage a clinician from choosing a DOAC for anticoagulation for an off-label indication. Recently, agents like idarucizumab and andexanet alfa were approved for reversal of dabigatran and apixaban/rivaroxaban, respectively [54]. Though there is a high cost associated with these agents and availability is extremely limited, this might improve in the future, and thus, may lead to a further increase in the use of these agents.

## Conclusions

At this point in time, it can be said that DOACs offer a promising alternative to traditional anticoagulants in patients with PVT with or without cirrhosis, but we need more information in the form of randomized controlled trials to guide us further. Most of the available data at present is from case reports/series and small retrospective studies. There is just one published randomized control trial that studies one DOAC agent (rivaroxaban) in the setting of acute PVT in patients with cirrhosis. We need more information that will guide us in selecting an appropriate DOAC agent in a particular setting of PVT as it is not a single uniform entity. Also, we need more clarity on the appropriate dosage, duration of DOACs, and the need for parenteral bridging anticoagulation in patients with PVT. 

## Appendices

Search strategies

PubMed Search Strategies

("Factor Xa Inhibitors"[Mesh] OR direct factor Xa inhibitors OR direct-acting oral anticoagulants OR direct-acting oral anticoagulants OR “direct oral anticoagulants” OR dabigatran OR Pradaxa OR rivaroxaban OR Xarelto OR apixaban OR Eliquis OR edoxaban OR lixiana OR DOAC OR “non-vitamin K antagonist” OR “factor II inhibitors” OR “oral direct thrombin inhibitor”) AND (“portal vein thrombosis” OR “portal vein thrombus” OR PVT)

Embase Search Strategy

('direct oral anticoagulant'/exp OR 'blood clotting factor 10a inhibitor'/exp OR 'blood clotting factor 10a inhibitor' OR 'blood clotting factor xa inhibitor' OR 'direct factor xa inhibitor' OR 'direct factor xa inhibitors' OR 'factor xa inhibitor' OR 'factor xa inhibitors' OR 'direct acting oral anticoagulant'/exp OR 'dabigatran'/exp OR 'dabigatran' OR ‘pradaxa’ OR 'rivaroxaban'/exp OR 'rivaroxaban' OR 'xarelto' OR 'apixaban'/exp OR 'apixaban' OR 'eliques' OR 'eliquis' OR 'edoxaban'/exp OR 'edoxaban' OR 'lixiana' OR 'non vitamin k antagonist oral anticoagulant'/exp OR 'factor ii inhibitors' OR ‘oral direct thrombin inhibitor’) AND ('portal vein thrombosis'/exp OR 'portal thrombosis' OR 'portal vein thrombosis' OR 'portal vein thrombus' OR 'porto thrombosis' OR 'pylethrombosis' OR 'thrombosis, portal vein' OR pvt) AND [embase]/lim NOT ([embase]/lim AND [medline]/lim)

Cochrane Central Search Strategy

(ZU "factor xa inhibitors" OR "factor Xa inhibitors" OR ZU "apixaban" OR apixaban OR Eliquis OR ZU "dabigatran" OR dabigatran OR pradaxa OR ZU "rivaroxaban" OR rivaroxaban OR xarelto OR ZU "edoxaban" OR edoxaban OR lixiana OR "factor II inhibitors" OR DOAC OR “oral direct thrombin inhibitor” OR otamixaban OR betrixaban OR bevyxxa) AND ("portal vein thrombosis" OR “portal vein thrombus OR PVT)
